# Senior Women’s Dance: From Pleasure to Emancipation

**DOI:** 10.3390/ijerph19106318

**Published:** 2022-05-23

**Authors:** Krzysztof Pezdek, Wojciech Doliński, Agnieszka Zygmont

**Affiliations:** 1 Department of Physical Education and Sport, Wroclaw University of Health and Sport Sciences, 51-612 Wroclaw, Poland; 2 Institute of Sociology, University of Wrocław, 50-137 Wroclaw, Poland; wojciech.dolinski2@uwr.edu.pl; 3 Department of Physiotherapy, Wroclaw University of Health and Sport Sciences, 51-612 Wroclaw, Poland; agnieszka.zygmont@awf.wroc.pl

**Keywords:** older adult women, dance, Habermas, qualitative research

## Abstract

The aim of this paper is to capture older adult women’s experience of dance. To this purpose, a qualitative research study was carried out with members of the ‘Gracje’ dance group. The study used Jürgen Habermas’s theory of communicative action as its theoretical underpinnings. The focus was on the models of action and validity claims expressed in language (narrative). In this theoretical framework, dancing activity has been shown as promoting not only physical health and mental wellbeing but also social involvement. Our study has found that, in and through dance, the older adults primarily realised their claims to pleasure, attractiveness, health and emancipation. This has considerably improved their bodily capacity and increased their self-esteem. However, what the older adults themselves find most important is that the realisation of these claims beneficially affects their interactions in family and neighbourly communities and facilitates their engagement in volunteer activities, helping people at risk of exclusion due to age and/or disability.

## 1. Introduction

This paper aims to capture the social role of older adult women’s dance. To this end, we carried out a qualitative study with the members of a women’s dance group called ‘Gracje’ (literally: the Graces; http://gracje.pl, accessed on 18 May 2022). (Importantly, the study of women’s situation in this part of Europe has a dual rationale to it. Firstly, older adults’—particularly women’s—dance has been one of the most robustly developing amateur and hobbyist activities in Poland since 2014 [1]. Secondly, a range of developments detrimentally affecting the social situation of Polish women, including elderly ones, has been observed in Poland since 2000. Although Poland favourably stands out against other European countries in having the highest proportion of women with a university education, Polish women are exposed to: (1) an excessive load of family- and work-related responsibilities, resulting in Polish women taking the least leisure time in Europe; (2) one of Europe’s highest risk of long-time unemployment; (3) Europe’s earliest exit from the labour market and (4) one of the lowest life-expectancy rates over 60 years of age. Poland is somewhat similar in these respects to Slovakia, Hungary, Romania, Bulgaria and the Baltic countries [2]). For its theoretical framework, our study built on Jűrgen Habermas’s theory of communicative action, where we focused on his concept of action models and validity claims expressed in language (narrative). In this theoretical perspective, dance proved a nuanced practice in terms not only of physical health and mental wellbeing but, above all, of social engagement. Our inquiry has found that dance helped the older adults realise primarily their claims to pleasure, attractiveness, health and emancipation. It has also revealed that these claims were realised through teleological, normative, dramaturgical and communicative models of action. Habermas’s theory of communicative action and our adjusted qualitative instrument made it possible to address and locate dance in a comprehensive spectrum of social interactions. We have shown that dance is a useful tool not only for improving people’s health status but also for promoting their engagement in new social relations, learning and handling new challenges that arise as they pursue their passion for dancing. Since Habermas’s theory of communicative action has not been used yet to explore dancing activity in the lives of older adult women, this study is supposed to redress this gap.

## 2. Theoretical Assumptions

As the elderly population is steadily growing, there is a marked interest in and emphasis on the social aspect of healthy ageing in the literature. Particular attention is being paid to the ageing of women since they are known to lose not only their capacity for motor activity but also their interest in it faster than men do [3]. Meanwhile, dance is one of the activities that help to sustain older adults’ social bonds [4]. If dance can substantially contribute to diagnosing and understanding changes that unfold in older adults’ bodies, personalities and relationships with others, dance can also be of key relevance in older adults’ adaptation to these changes [5,6,7,8]. Dance furnishes them with ample opportunities for highlighting their attractiveness and for being perceived by others as attractive and open to establishing new social relations [9]. Older adults’ active involvement in dance groups enhances their sense of community belonging and fosters their members’ mutual understanding [10,11]. Besides, it motivates individuals to develop new interests and to take part in other activities, ones as varied as painting classes and Esperanto courses [12,13,14,15,16,17,18,19,20,21]. As such, dance productively binds together older adults’ individual and communal functioning as it enhances their biological outcomes (for example, by strengthening the musculoskeletal system, improving the cardio-respiratory capacity and reducing the risk of metabolic disorders) and socio-cultural wellbeing alike [1,3,4,15,16,17,18,20,22,23,24,25,26,27]. Addressing and promoting the processes that help to more effectively tap into elderly people’s social capital is a civilisational challenge today. This provides the background against which the explorations of social theorists and philosophers [28,29,30,31]—in particular Habermas—prove highly pertinent and universal.

According to Habermas, the world has a three-fold ontological structure that is composed of objective facts (states of affairs), social norms (morality) and subjective experiences (moods, desires, feelings). Each of these components is correlated with at least one model of action and its corresponding way of expressing validity claims in language.

We realise that, in his Theory of Communicative Action, Habermas also explored the philosophy of language, mostly investigating Gottlob Frege’s universal pragmatics and the theory of speech acts developed by John L. Austin and John Searle [32,33]. Habermas was particularly preoccupied with the interactional constitution of validity and meaning. However, in this article, we do not address these issues as our primary focus is on the implications of validity claims expressed in language and their corresponding models of action.

The teleological (strategic) model of action is cognitive and is used by individuals to explain the meaning of the objective world or to pursue concrete aims [32]. As truth validity claims are raised in this model, it can be described in true statements. The normative model of action is both cognitive and motivational. Based on the normative action model, individuals produce propositions that make sense of the social world. In these propositions, they test whether motives and actions are consistent with or whether they contest the norms in place [32]. Hence, individuals raise validity claims concerning social norms in this model, which is expressed in ought-sentences in language. For its part, the dramaturgical action model reflects individuals’ subjective experiences, and it can be expressed in statements about desires and feeling [32]. Individuals can rely on dramaturgical action to communicate their sincere emotions and wishes to their audience. However, one can also seek to produce false impressions by expressing insincere feelings, and, on such occasions, one enacts a particular role, at the same time concealing one’s real experiences. The dramaturgical action model is the basis for raising validity claims about intentions and serves to determine whether one can attain one’s teleological (strategic) goals while maintaining the truthfulness of one’s experiences (desires and feelings). Finally, the model of communicative action is geared to achieving understanding (consensus). As compared with the other models, communicative action involves the most extensive use of language. This is due to the fact that the speaker and the listener simultaneously refer to a common ground, which may be provided by some state of affairs, social norm or moral value or a subjective experience in order to negotiate a shared definition of the situation. This jointly established definition will later provide a plane for further negotiations [32]. In the communicative action model, validity claims are raised regarding all the other three action models at the same time [32].

By relying on Habermas’s theory, our study has yielded a comprehensive account of the older adult women’s passion for dance, in which a specific practice expressed in action models is fused with narratives about this practice as conveyed in language-based claims to truth, legitimacy of norms and truthfulness of feelings.

Although Habermas’s original considerations primarily concerned written texts, his framework can be effectively applied to interpret speech [34,35]. In our study, analyses of the respondents’ utterances helped us to identify the ways in which they reached a narrative consensus based on what Habermas referred to as the intersubjective recognition of validity claims [32]. They revealed what the older adults had thought and experienced in particular dance-related situations against the background of cognitive, moral and expressive elements of their cultural knowledge [32]. The older adult dancers freely and unconstrainedly formulated particular claims as true/untrue, recognised particular social norms as right/wrong and expressed particular experiences as sincere/insincere [32]. The participants’ easy and uninhibited attitude to cultural knowledge not only fostered their self-understanding as dancers but was also a basic factor in their cooperation, which generated their group identity as ‘Gracjanki,’ that is, the members of ‘Gracje’ [33].

## 3. Research Methodology

Consistent with our theoretical framework, we selected the older adult women’s dance group by means of purposive sampling and based our research on an adjusted strategy of qualitative interview [36,37]. We applied the semi-structured life world interview [38], whose script, which contained research questions and second questions prompted by the older adults’ narratives, relied on the strategy of narrative interview and, to some extent, conceptual interview [36]. This procedure ensured that the participants could talk freely and extensively and stop or interrupt whenever they felt like doing so, without being afraid of criticism or negative assessment from the research team [35,39,40,41]. In this way, we adhered to the ethical norms of social research [42].

Three researchers conducted interviews with the ‘Gracje’ dancers, each on the same day (thirteen interviews in total, ranging from 56 to 129 min). The interviews were carried out at a venue the dancers had themselves selected as a peaceful and secure setting (the University of Health and Sport Sciences in Wrocław). Having previously been there to attend practical courses (yoga, healthy-spine sessions), they knew the place well. Because of the restrictions caused by the COVID-19 pandemic, some of the interviews were remotely administered (on Zoom). The friendly atmosphere was promoted by the fact that the research team included a coordinator of volunteer projects in which the participants were involved. Their shared good experiences and mutual trust provided a platform of understanding throughout the research process (helpful in initiating meetings, conducting the interviews and explaining non-verbal responses). The interdisciplinary research team developed an interview script comprising the following research areas: (1) practice sessions, the selection of the repertoire and preparations for performances; (2) the body, clothes, props and emotions; (3) relations among the dancers and (4) relationships between the dancers and their social environment (family, friends, neighbours, older adults’ organisations, audiences, juries and sponsors). In all these areas, the researchers sought to identify the respondents’ dance-related experiences that could reveal social transformations unfolding across various domains (family, generations, work, stereotypes, etc.) over the last few decades.

The jointly performed transcription of the interviews with the participants and multiple listenings to the audio recordings made for the researchers’ in-depth exposure to the data. Team discussions, which were considerably aided by one member’s practical knowledge of dance, helped collectively develop, first, a list of analytic codes and, then, categorisation schemes [43]. In the subsequent interpretation phase [44], those, first of all, made it possible to avoid quoting the respondents merely as a result of the questions asked in the interviews [41,45,46] and, secondly, prevented the researchers from excessive attachment to their research assumptions and prior knowledge from before the study [47]. These effects were particularly supported by one of the elements of triangulation, specifically by the analysis of an earlier interview with the group’s dancing instructor. Her observations and assessment of the dancers’ situation prompted the researchers to build coding trees and interpret the data in a more reflexive manner [44,47]. Besides, a connection emerged between Habermas’s conception and the participants’ narrative approach to social changes occurring over the last few decades. This link took the form of claims to pleasure, attractiveness, health and emancipation [48,49].

The mean age of the older adult dancers in our sample was 71 years. All the participants lived in a city with a population of more than 500,000 inhabitants and had been retired for 12 years on average at the time of the study. One of the respondents was childless, two had one child each and the remaining ten had two children each. Six of the women ran their households alone, and seven of them with their husbands or partners. In 2022, two of the women quit the dance group, and two new dancers (aged 65 and 66) joined in their place.

The Table 1 below lists respective validity claims with their corresponding themes and key quotations from the interviews with our older adult participants.

## 4. Dance and Validity Claims: The Claim to Pleasure

### 4.1. Performing at Integrational and Festive Events

The respondents contrast performing at integrational and festive events with competitive dance. They definitely prefer the former because their aim is to show ‘that you can have a good time together and make the audience join in the fun. And this is really very pleasant’. Such performances are, first of all, about triggering positive emotions enjoyed by both the dancers and the audience [12,15].

The bottom line of such appearances is that they are not formally judged. Consequently, mistakes in dance routines, wardrobe malfunctions or imperfect make-up do not matter as much as in performances at contests. In fact, dancing blunders may even prove to be productive in integrating the audience and the dancers:

*There was an Independence Day event. And we performed there and wanted to do an integrative dance, telling the public how to dance. This dance is about moving in a circle, stepping out and stepping in. And the girls messed it up so bad that the host had to help them out. It was total bedlam. And what we’d wanted to show ended up really lame. Everybody was having a good laugh though.*

The older adults mostly give their integrational and festive shows at nursing homes, care homes, child-care facilities, kindergartens and various festivals:

*We perform at the Adult Day-Care Home. We dance for the clients, but also encourage them to dance with us. We danced our routine and then each of us approached a client that could walk and fancied dancing. And you danced with such a person, well, it was the way it was, but they were very glad. And we were glad, too, to have amused and helped them. These are completely different emotions, because these people are a bit disabled, but they like dancing very much, and they like having somebody come and visit them.*

This suggests that, in their performances at integrational and festive events, ‘Gracje’ undertake a helping mission, and their supreme aim is to show that movement means health and dance means happiness [19].

### 4.2. Competitive Dance

Nonetheless, ‘Gracje’ sometimes also take part in dancing competitions. Importantly, they have won some awards and distinctions; they have also been invited to participate in festivals held by Polish Radio. Yet, the older adults are not eager to perform on such occasions. They cite a few reasons for their reluctance. Firstly, they do not like being assessed by juries because they are perfectly aware of their imperfections caused by their age, less supple bodies and less agile movements. They also realise that competitive dance routines must be more complex and technically difficult, which is bound up with an increased likelihood of errors, stage-fear and stress: ‘it was a typical dancing contest. Emotions ran high, stress, flushed faces and general agitation. I guess it cost us a bit too much. And expectations too, because we felt we were good, and here there were all the experts and their calls. We knew our shortcomings, mistakes, because those always happen when you’re on the stage, but still we’d expected something more’. Secondly, competitive dance demands more vivid and technically correct movements, which turns dancing into sporting rivalry. This compromises some feminine features in dance as the respondents associate femininity ‘with something gentle, smooth, toned-down,’ ‘romantic, soothing and intimate’. Meanwhile, sharp, bold and rapid movements in dance may express something negative (e.g., anger) and jeopardise health (e.g., injuries). Thirdly, the participants are not keen to perform at contests because each of them takes personal responsibility for the entire group at such evets. Hence, mistakes made by individual dancers affect the evaluation of the performance of the group as a whole. For this reason, the seniors definitely prefer integrational and festive performances, in which their individual skills matter less than transmitting joy through dance and encouraging the audience to join in the fun. Fourthly, the respondents tend to avoid competitions because those are often held in venues remote from where they live, which entails parting with their families and incurring additional costs. The participants usually have to cover travel, food and accommodation expenses, buy new outfits and pay for extra practice sessions. Consequently, they have instituted a selection procedure for events to attend: ‘to decide where we go is up to our manager. She takes into account how far it is from Wroclaw, whether they offer transportation, whether there’s a meal option, etc.’.

## 5. Dance and Validity Claims: The Claim to Attractiveness

According to our interlocutors, dance should ‘release a woman’s soul,’ yet this releasing of ‘a woman’s soul’ took time and was incremental. At the beginning, each older adult had to train her body into a proper posture and dancing movements because ‘I’d slouch, and what with hunching and cowering, I expressed some kind of insecurity. And this movement [of mine] was not fluid, but curt in a way’. All this was connected to the women’s lack of self-confidence as dancers, which hampered the display of the attractiveness and sensuality of their bodies [13]. Our respondents only managed to overcome these restraints through dance in late adulthood: ‘when I started to believe that I could do it, that I felt better, was coordinated, fell into place in these movements, these steps, then I also noticed my femininity of sorts: hand motions, posture, head up, chest to the fore’.

### 5.1. Clothes, Make-Up and the Repertoire

Besides learning proper comportment and movements in dance, the imperative of highlighting one’s attractiveness through outfit, make-up and suitable repertoire selection was another important factor. Costumes are of considerable importance because ‘we aren’t young. Others may put on anything and look good, because they’re young. And we, unfortunately, must give it a thought to look good’. At the beginning, the dancers were advised to perform in uniform ‘Senior’ text t-shirts, but they found this idea unacceptable for two reasons. One was that this apparel did not express their self-perceived attractiveness, and the other was that it did not convey their mindset, which considerably differed from the conventional image of an older adult who most values family time and taking care of grandchildren. As a result of involvement in dancing, chronological age ceased to be a criterion in the respondents’ perception of self, possibilities of pursuing passions and wellbeing [22] because they discovered that one could lose oneself in dance and, as it were, enter another plane of reality: ‘dance helps me shut the world off. Then I feel relaxed, cooled out, happy’.

### 5.2. The Dancers’ Attractiveness

The dancers emphasise that what matters in the group is not only the personal attractiveness of individual dancers but also the attractiveness of the group as a whole. This is of particular importance at competition appearances, where the coherence of the group is judged not only in terms of the repertoire it puts together but also in terms of alluring visual presentation. In this respect, costumes unify the group and imbue it with stylistic and visual uniformity. The appeal of performances also hinges on the proper choice of the colour of outfits: ‘with these red skirts on, we are so feminine and show so much love though our clothes; we felt very feminine. Such a real woman! A woman. And those movements, too. Matching black gloves, roses, black shoes and red dresses to boot. And we felt so attractive and light’.

Aware of their bodies, the dancers seek to realise their claims to attractiveness through dance. They do so in a very deliberate and purposive way. They know what body posture to adopt, how to move, what garments to wear and what accessories to use. They know which body parts to expose and which to hide: ‘One must disguise her spare tyre, another something else. Obviously, like women, one wants to cover her legs, and another wants to show them’. They advise each other on make-up and hairstyle. While they have no professional visagiste, they rely on their own experience to design their make-up so that they match and complement their dancing repertoire. Still, what matters most is that all the women should feel good and derive pleasure from dancing, whether they perform at a contest or at a festive event: ‘Women like being admired, and whether it’s for their clothes or for their good dancing doesn’t make a difference’.

### 5.3. Stereotypes

Our respondents stress that their behaviour breaks stereotyped representations of elderly women as crooked, with dishevelled hair and shabbily dressed. Because stereotypical elderly women spend their leisure time at home, their lifeworlds shrink for the dearth of sharing thoughts and views with other people [50]. They feel underappreciated, become bitter and feel bad about themselves. Such attitudes may result in the marginalisation of elderly people and even in their exclusion from the practices of social life. As opposed to that, the women in our study like going out and meeting other people. Additionally, their self-esteem has considerably increased: ‘when I look nice, and people like me, I am more feminine and attractive to men’. They explicitly depict themselves in terms of attractiveness: ‘we’re all sexy now’. They are not afraid of marginalisation or exclusion from social interactions because their involvement in ‘Gracje’ has bolstered their pro-activeness: ‘a woman’s worth is increased by going to practice, and we go to practice a lot’.

## 6. Dance and Validity Claims: The Claim to Health

The older adults in our study have endeavoured to take care of their health since a young age. They defined health in terms of physical activity (fitness), diet and wellbeing. They basically did not address health from a strictly medical perspective because they believed that ‘a pill will not replace exercise, but exercise will replace a pill’. Hence, they cited a range of exercises they found important: they loved cycling, practised Nordic-walking, went swimming, walked a lot, tended gardens and took part in training courses focused on a healthy spine, proper posture and diet, including those offered by the University of the Third Age (U3A) at the University of Health and Sport Sciences in Wrocław (Polish: Akademia Wychowania Fizycznego, AWF). At the same time, they recognised limits to pro-health activities: ‘We can put in some work, but generally the genes are responsible for one’s looks and lifestyle’.

However, the women were aware that increased bone brittleness was a significant factor in limiting the physical activity of elderly people: ‘the girls easily fold, trip over, and it’s a problem. You slip on a pebble, and harm’s done. Once you could keep your balance, and now you tumble’. Maintaining the proper body mass also became a challenge: ‘Over the climacteric, I put on ten kilos. Not so much putting on weight, as rather swelling, I was huge. I hated my body!’

### 6.1. Physical Exercise and Fitness

The dancing practice was well correlated with the seniors’ claims to health because it afforded them opportunities for physical exercise: ‘dance as gymnastics, only better and more fun’. Dancing increased their physical strength and fitness, improved their motor coordination and made them feel ‘light when walking,’; they even confessed that ‘but for that we’d be crippled by now’. At the same time, the women are not uncritical about dance because they know the constraints imposed by their ageing bodies. Consequently, when a routine designed by their instructor seemed too risky (causing a health hazard) to them, they insisted that it be revised. However, the instructor sometimes proposed exercises that occasioned the dancers considerable difficulty: ‘There was a situation once that we knelt down. And now, let’s stand up beautifully. I go: well, the instructor is fifty, and we—hardly any is younger than seventy; ok, ok, it was fun twenty years ago’. Yet, such occurrences were relatively rare since the exercises for practice sessions were, as a rule, sequenced according to their difficulty, from the simplest to the more complicated ones that required more agility or effort: ‘it starts from a short gym session, and then stronger exercises follow’.

### 6.2. Diet

Dance not only offers the older adults an opportunity for exercise but also motivates them to be mindful of their diet [18]. Even though they realise that diet is relevant to health, they are not restrictive about it: ‘I’m not good with diets. All my life I tried one diet or another, but now I’m easy on myself because at my age anything goes’. The respondents’ dominant attitude involves relativising their diet to fit their own needs: ‘diet is good, but, well, I love my food, it’s not even about pastries, it’s about different things. I’ve stopped eating meat, though, not entirely in fact; still, 90% of my diet is composed of fruit, veggies and cereals’. While the women state that they are interested in publications on healthy nutrition, their actions in this respect are guided by general rules: ‘I try, so to say, not sparkling, unsweetened, unprocessed, you know, such overall recommendations’.

### 6.3. Mind

The women in our study pointed out the salutary influence of dance not only on the body but also on the mind. Dance became an important source of satisfaction, joy and gratification from life for them. Besides, it made them more receptive and self-confident in interpersonal contacts. The change is visible in the way they talk with others because they no longer ‘keep their eyes down’ and their facial expressions are more ‘genial’. However, to be able to fully enjoy dancing, they first had to shake off physical constraints because the mind is expressed through the body: ‘This need to dance comes from mental and emotional needs, and this is expressed in physicality. If I become unblocked and dance, whether freely or even doing a routine, I’m powered up, so to speak’.

### 6.4. Joy

Though physically exhausting, dancing and proper practice engender a feeling of bliss that is somewhat reminiscent of a state following alcohol consumption: ‘I’m emotional, I’m happy that I can dance to the rhythm of music, and it gives me so much pleasure as if I’d had a sip of wine, though I don’t drink’. The discharge of endorphins induces joy and good mood not only in the dancers themselves but also in their friends and loved ones: ‘the practice is over, one goes back home, to one’s kids, or one goes out with a friend to have coffee or pizza, and all this comes out in these intercourses. This joy, these endorphins come out. You hear, for example: How come you’re so radiant today, or you look so pretty, what’s up? It’s nothing on the face of it, but apparently, it’s there in the sparks in your eyes’. The joy released in dancing helps the respondents reduce bad emotions and forget about everyday problems: ‘Even the daily gripes, what with these constant nuisances and shortcomings, I simply forget about them. And if there’s practice twice a week, nothing aches me sometimes. I’m relaxed and happy to be dancing’.

## 7. Dance and Validity Claims: The Claim to Emancipation

The claim to emancipation (liberation) is another claim frequently articulated by our respondents in the interviews. This claim appears to be seminal because it is a composite of all the other claims depicted above. Actually, the claims to pleasure, attractiveness and health become truly vivid and meaningful in aspirations to emancipation. The participants associate emancipation with social, mental and moral contexts.

### 7.1. Social Emancipation

In the social context, the respondents describe emancipation as contesting the model in which women (especially elderly ones) have very particular roles in family and the community as a whole ascribed to them, specifically involving caring and educational functions, household chores (e.g., laundry, cooking, shopping, etc.) and spending free time at home amidst family (with the husband and/or children). According to the dominant model, these activities and engagements should fill women’s time, and this cultural framework was opposed by the older adults in our study, who made their claims to emancipation in relation to it. This was by no means easy because the model was firmly entrenched in social relations and handed down from generation to generation with only minor modifications: ‘When I joined the dancing classes, my mum was still alive, she was ancient and would say: “How do you mean? You? Unheard of. You should knit, make dumplings and read fables to the children”’. That course of things was taken for granted by our respondents’ mothers, who considered dancing an extravagant whim and an aberration. Consequently, the mothers tried to discourage their daughters, themselves older adults, from pursuing such interests and recommended cultivating the tradition, which they believed warranted order and security in family and social relationships.

Ambivalent social responses also often stem from the fact that young families grapple with steep demands of the housing market, which force parents to work extra hours to be able to take out home loans and pay monthly interest (not infrequently for dozens of years). With a shortage of places in creches and kindergartens, parents must arrange day-care for their children on their own and often believe that they are justified to expect older family members to help them out by looking after their grandchildren. While our respondents understand these difficulties, they also perceive some ‘abuse’ from their children, who forget that their mothers also have a right to enjoy their passions and to spend time outside of the family circle: ‘most women are burdened with their grandchildren, but have no guts to tell their children that they’d also like to do something for themselves. Life may indeed be hard for the young, because work demands engagement round the clock, more than when we worked, but at the same time, they mustn’t exploit their mothers’.

The respondents obtained scarce support from friends and acquaintances, especially at the beginning of their adventure with ‘Gracje’. People they knew could see no point in spending leisure time in this way: ‘whenever I told my friends I was going to practice, they’d say: “You must be out of your mind! What’s in it for you now you’re old?” I was simply criticised’.

Whereas criticism of the participants’ dancing passion was commonly voiced by their friends and loved ones early on, as time went by, the group of their supporters grew. More than that, their number increased along with the respondents’ commitment to dancing and their dedication to promoting dance as a pastime for the elderly. When their families concluded that their mothers’ and grandmothers’ claims to dancing activity were true, morally justified and sincere, they began to support their passion and to adjust their own expectations of the seniors to it: ‘My children find it a good thing. My granddaughters say that granny is super, cool! They’re happy. My daughter says: “Mum, it’s great that you’re not stuck at home but do something”’. Even the seniors’ ancient mothers, as a rule, changed their attitude to their daughters’ passion as soon as they saw them perform: ‘I had to overcome my mum’s mental barriers, which were not insignificant; she was a child of her time, and she found it difficult to understand that a no-longer-young woman could have her whims. But when there was a show of dancing in a circle on a lawn on our estate, my mum limped over, sat down, watched us and said: ‘My, my, it’s cute!’

### 7.2. Mental Emancipation

Our interlocutors admit that they found mental emancipation the greatest challenge. The dominant social model of a woman was ‘etched’ in their minds so deep that, when things changed enough to contest it, the older adults from our study had quite considerable misgivings about it. They were not certain whether their claims to dancing, meeting up with their women-friends and going to festivals and competitions together were right and whether their realisation should be negotiated. Still, developing self-belief was the hardest thing for them: ‘My prior insecurity began to morph into self-confidence. Confidence in my worth. Because once, when I was a young girl, I got to hear many things at home that made me feel insecure. And it was in dancing that I started to develop self-confidence. When dancing, I’m self-confident. When I was dancing, I could express this self-confidence, this satisfaction with life, the fact that I wasn’t a person who had nothing to show for herself’. Dance was a therapy that helped the women acquire self-confidence and self-belief: ‘It’s therapy for me. Once I went to sessions with a psychologist, now I don’t need that’. When recounting their mental emancipation, the respondents most frequently used expressions such as ‘I opened up’, ‘I went out of my shell’, ‘I started to talk and communicate with people more’ and ‘I began to go out more often’.

The respondents’ mental emancipation contributed to their repudiation of the dominant social model of an elderly person. The women in our study agree that ‘the elderly can be active, have lives of their own and don’t have to lean on anybody. Of course, they may need help from time to time, but they can also offer help to others. Their lives don’t have to be associated with life as it once was, only going for a walk, picking up grandchildren and constantly worrying whether or not they’ve done their homework, whether or not they’ve got an A’. They are rather inclined to admit that old age is less linked to one’s chronological age and more to one’s mindset: ‘youth is in the head, you can train it’. Therefore, one should not be afraid to engage in relations with others, whatever their age. According to the dancers, it is not true that the young are negatively predisposed against the elderly. One woman cited her own experience as evidence: ‘I went to several dance schools in Wrocław; they’re full of young girls, lower secondary school students by the look of them. But I had nothing against it. Indeed, most young people are not negatively disposed vis-à-vis the elderly. At least, I felt nothing of the kind’.

Nonetheless, the participants admit that many elderly people enact stereotypes of old age. They stop taking care of how they look, do not look for new opportunities of self-realisation, tend to perceive the world in negative terms and are dependent on their families: ‘Some ladies look really shabby. I don’t want to be critical. There’s a difference in the looks, responses to the world and people. A huge difference. I know this, having talked with my friends who do nothing. They’ve simply been labelled in this way, and they can’t open up their minds, it’s tunnel vision of sorts’.

### 7.3. Moral Emancipation

According to our interlocutors, dance is instrumental for overthrowing several stereotypes about older adults, and, for this reason, they treat their passion not only as a source of personal pleasure, attractiveness and health but also as a mission. Its goal is to emancipate the elderly, as well as other people at risk of exclusion from social practices, by encouraging them to abandon a passive attitude to life and advocating for every individual’s right to autonomy and development: ‘you can take up something new, no matter how old you are. And, for me, dance is this thing’.

Before the members of ‘Gracje’ could stand up for respecting the human rights of all people, whatever their age, health status, education, etc., they first had to experience a moral emancipation, which resulted, among other things, in making claims to pleasure, attractiveness and health. They also needed to develop a belief that it was worthwhile to, at least partly, emancipate themselves from the family duties put on them: ‘“Gracje” became a support group. And we started to liberate ourselves a bit from all those kinds of domestic limitations. We help each other. We talk’. Their self-perceived attractiveness significantly increased. The older adults consciously began to underscore the beauty of their bodies, insisting that looks were important for all women, irrespective of their age: ‘That’s this age group where femininity was inadvisable and even had to be concealed. And here we learn that showing your femininity is positive and beautiful. And this is what we can teach the next generations, our daughters and granddaughters, for them to learn to express themselves through movement’. Beauty and the sense of attractiveness are important not only in practice sessions and performances but also in common everyday dealings: ‘When I dress up and go to Wrocław, I feel more elegant. At home, you don’t tend to dress up. Though I pay more attention to it now’.

The attitude and activity of our respondents inspire admiration in other people who have neither taken such passions and related emancipatory pursuits for granted nor deemed them unambiguously commendable before: ‘How lovely this looks, how amazing that you can do it, that age doesn’t matter when dance is concerned. It doesn’t matter, this is beyond fantastic’. Such comments only confirm that our respondents’ stance is right and that it provides motivation for further action aimed at emancipating the self and others: ‘Everybody must have their passions and the meaning of life in order to be able to seek self-realisation later, in late adulthood, rather than expecting others to take care of them. You need to work on this for old age, late adulthood, to be nice for you and for people around you’.

## 8. Conclusions

Drawing on the models of action proposed by Habermas, we have examined the circumstances under which the older adult women raised validity claims expressed in their narratives about dance. Their claims should be viewed as exemplifying the pursuit of universal aspirations as they included claims to pleasure, attractiveness, health and emancipation. Our framework has helped us to explore dance as a practice of action, along with reflection on this practice as embedded in various settings of elderly people’s lives. This involved not only individuals’ attempts at satisfying their personal needs for pleasure, attractiveness, health and emancipation but also efforts to meet social needs: belonging to a group, contributing to its identity, performing for an audience, helping people at risk of marginalisation, spending time with friends and promoting the human right to self-fulfilment.

Our older adult respondents’ dancing adventure began from their search for a satisfying course in the various programmes offered by the organisations and institutions in place. Having found that there was no suitable dancing class for them to enrol in [1], they founded ‘Gracje’ as their own dancing group. From the perspective of Habermas’s theory of communicative action, this proves that their validity claims were true (i.e., the participants correctly defined the initial situation—facts and states of affairs), that they were morally legitimate (i.e., the women justified their moral right to satisfy their needs in and through dancing) and that they were truthful (i.e., the respondents made their social environment understand and approve of their own feelings and wishes related to dancing passion).

Our study has found that the older adult dancers primarily realised their claims to pleasure in dramaturgical action, where the expression of subjective feelings was the central factor [32]. In this case, claims to—their own and the audience’s—pleasure were what mattered most to them. Hence, although they were proud to talk about the awards and distinctions they had won in various contests, they still associated these successes with excessive stress and inevitable expenditures. That is why, in their teleological action, they tended to decline invitations to competitions and opted for performances at care homes or local festivals. In the latter, the correct completion of a pre-arranged routine and rivalry with other groups were secondary concerns, and occasioning pleasure to the elderly and people with disabilities was the women’s pre-eminent object. This claim was also realised in norm-regulated (moral) action because the older adults put eliciting positive emotions in clients of nursing homes above being appreciated by competition juries. Therefore, they chose dance as a mode of helping people in need rather than dance for the sake of rivalry, formal assessment and personal sense of achievement. Nonetheless, the dancers always negotiated the choice of performance with each other in communicative action, and, at times, the consensus they reached involved delegating a smaller number of dancers (for example, six) who decided to take part in a contest and were ready to cover the expenses incurred.

Like their claims to pleasure, the respondents predominantly realised their claims to attractiveness in dramaturgical action [32]. In and through dance, they could take a position in their own subjective worlds and discover in them claims to being beautiful, admired, sleek, content and sexy. Dramaturgical action helped the dancers not only to identify these claims but also negotiate their realisation in communicative action. This resulted in practising the proper body posture, the selection of flattering outfits and accessories and the arrangement of suitable repertoires and routines, with all this being perfectly aligned with teleological action. Whatever the older adults undertook, their projects were verified by the feedback from their audiences and, more broadly, from society, because the dancers also exhibited their enjoyment of life in daily comings and goings.

As to claims to health, the participants, first and foremost, realised those in teleological action. Consistent with the objective state of affairs, they referenced and took into account ageing and the role of physical exercise in the process. They rationally tested a range of dance routines to select those that best corresponded to their needs. On each such occasion, they also paid attention to the limitations resulting from their age and dancing skills. Additionally, we have found that the older adult women’s claims to health were normatively reinforced since the respondents emphasised that their health affected not only their own contentment but also that of other community members, e.g., neighbours [51]. Crucially, when engaging in norm-regulated (moral) action, the women often contested the social models in place, which generally barred the elderly from making claims to the pursuit of their passions (particularly new ones).

Our findings indicate that the older adults’ claims to pleasure, attractiveness and health took on vivid significance in the context of emancipation. Habermas understood emancipation as liberation from a dogmatic dependence, as unfettered (symmetrical) communication and as dominance-free dialogue [35,40]. The members of ‘Gracje’ confirmed in their interviews that their claims had often been dogmatically treated by their interlocutors. On such occasions, the women had striven to achieve consensus in the communicative process, with consensus understood, first, as the recognition of their need to practise dancing and, second, as not impeding their involvement in this activity. Above all, they had endeavoured to emancipate themselves from the dogmatic model of old age as a period in human life in which the experience of pleasure, the aspiration to be attractive, the effort to retain good health and the desire to spend leisure time with one’s friends (rather than with one’s family) should be irrelevant.

Hence, the realisation of claims to emancipation was premised on the older adult women’s considerable social engagement. Consequently, our respondents addressed the issue of respect for human rights, especially regarding people at risk of exclusion from social practices. In communicative action, the dancers negotiated shows primarily intended for the elderly and people with disabilities. They also performed at events that involved public-consciousness-raising about age- and/or disability-related social exclusion and promoted dance as a tool for interventions targeting individuals (groups) at risk of social marginalisation. Involved in this way, the participants became ambassadors of dance, promulgating its socialising, therapeutic, health-related and, above all, expressive values.

The older adult dancers’ claims to emancipation comprised all of Habermas’s models of action, although communicative action appears to have prevailed as it served the respondents to negotiate their own good (claims to pleasure, attractiveness, health and emancipation) and the good of others, particularly of marginalised individuals and groups, with various people and institutions.

The benefits of dance we have shown are consistent with other authors’ findings, although our older adult respondents exhibited an exceptional interpretive originality in addressing the issues under discussion. As a result of involvement in dancing, the mindsets of these older adults were characterised by greater openness, self-confidence, spontaneity and joy of life. This translated into their increased self-esteem [12,14,18,22], which was additionally buttressed by improved fitness, nimbleness and motor coordination [14,18,23]. Our research implies that the participants predominantly attempted to realise their claims to health through physical exercise and bolstering intra-community bonds (family, friends) rather than by strictly medical means. They had reservations about the public healthcare system, and, if their condition did not demand a physician’s intervention, they sought to remedy their physical and mental complaints on their own.

Besides, our study suggests that the respondents built on their positive feelings and desires released through dance in other social settings [13]. They became more self-reliant, independent and receptive to enjoyment in everyday life. They actively looked for new interests, which they were eager to share with their families, friends and strangers. The older adults enrolled in various programmes and courses (painting, cooking, fashion, Esperanto, etc.), often ones offered by the U3A and what are called in Poland seniors’ clubs. They also engaged in volunteer work at hospitals, care and education facilities for youngsters, nursing homes, etc. Shows for such audiences often made them give up on participating in dance contests and competing with other groups. This was a moral choice because helping others was what the dancers valued more than their other commitments.

The older adults in our study explicitly stated that involvement in dancing had improved their skills of conflict-solving, collaborating with others and assessing everyday intra-group relations. This was useful in family life (conflicts with husbands, children and grandchildren), cooperation with fellow dancers (negotiating the selection of the repertoire, outfits, organisation of performances and trips) and relations with other people (organisation of charity events, negotiating the hire of an instructor, renting the practice room). The older adult dancers’ openness to collaborations and their perception of their own weaknesses related, among others, to ageing (increasingly fragile bones, diminished suppleness of the body) as surmountable challenges helped them to overcome multiple stereotypical constraints and instil emancipatory aspirations in others, especially in people at risk of exclusion due to disability, homelessness and/or age [12,13,14,15,18,19].

Notably, dance also increased the respondents’ self-satisfaction and made them feel more attractive [13]. Interestingly, the participants were capable of transferring their claim to attractiveness onto other people, which was particularly exemplified in their relations with the audience [15]. During their shows, they time and again kindled self-perceived attractiveness not only in other older adults but also in people with disabilities [12]. The dancers’ integration with their viewers fostered the sense of integral belonging to a community in all the participants. As a consequence, the feelings of loneliness, which are so common among the elderly, were reduced [18,52]. In this way, the women act as ambassadors of dance [19].

In their interviews, our respondents repeatedly emphasised that, in and through dance, chronological age had ceased to be a criterion in their self-perception. They shook off shame, fear and prejudice, discovering that, as they were growing older, they encountered new opportunities for self-expression and pursuit of their interests. They were no longer afraid to underscore the attractiveness of their bodies through clothes, make-up and, first and foremost, comportment (heads up, chests to the fore) [22]. This shows that dance is not only a physical activity that may ameliorate the process of ageing [22] but also a factor in encouraging a healthy diet and attractive appearance. The attitude to diet reported by the older adults in our study was intriguing. While the respondents realised that they should eliminate some products from their diet, they often refused to do so, explaining that they were already allowed not to be slender. Consequently, they tried to observe general principles of proper nutrition but avoided diets that would force them to alter their culinary preferences or lifestyles.

Although our research is consistent with international research findings [9,10,11,53,54,55,56,57], given its east-central European situatedness, it should be expanded by studies of other dance groups. The scarcity of corresponding representative studies of Poland precludes any valid statistical comparison with the situation of older adults before 1989 [58,59]. At the same time, the connection between transformations of social institutions providing services for older adult women and the development of older adult women’s consciousness, competencies and activity types suggests that the changes have been unique, profound and qualitative [1,60]. The strong contribution of this paper is that it applies Habermas’s theory of communicative action to explore this multidimensional interplay. Habermas’s framework has been instrumental in revealing the strong interconnectedness of action and language (narrative), which has become vigorously meaningful in the older adult dancers’ emancipatory aspirations.

## Figures and Tables

**Table 1:** Validity claims with their corresponding themes and key quotations.

Validity Claims	Themes	Key Statements
The Claim to Pleasure	Performing at integrational and festive events	You can have a good time together and make the audience join in the fun. And this is really very pleasant.
	Competitive dance	I guess it cost us a bit too much.
The Claim to Attractiveness	Clothes, make-up and the repertoire	We are so feminine and show so much love through our clothes
	The dancers’ attractiveness	Women like being admired, and whether it’s for their clothes or for their good dancing doesn’t make a difference
	Stereotypes	When I look nice, and people like me, I am more feminine and attractive to men.
The Claim to Health	Physical exercise and fitness	A pill will not replace exercise, but exercise will replace a pill.
	Diet	All my life I tried one diet or another, but now I’m easy on myself because at my age anything goes.
	Mind	If I become unblocked and dance, whether freely or even doing a routine, I’m powered up.
	Joy	This joy, these endorphins come out. You hear, for example: How come you’re so radiant today, or you look so pretty, what’s up?
The Claim to Emancipation	Social emancipation	Most women are burdened with their grandchildren, but have no guts to tell their children that they’d also like to do something for themselves.
	Mental emancipation	When dancing, I’m self-confident.
	Moral emancipation	That’s this age group where femininity was inadvisable and even had to be concealed. And here we learn that showing your femininity is positive and beautiful. And this is what we can teach the next generations.

## Data Availability

The data presented in this study are available on request from the corresponding author. The data are not publicly available due to privacy restrictions.
